# Trends in service time of pacemakers in the Netherlands: a long-term nationwide follow-up study

**DOI:** 10.1007/s12471-017-1024-x

**Published:** 2017-08-02

**Authors:** L. M. de Vries, M. J. G. Leening, W. A. Dijk, C. A. M. Hooijschuur, B. H. C. Stricker, N. M. van Hemel

**Affiliations:** 1 Department of Epidemiology, Erasmus MC – University Medical Center Rotterdam, Rotterdam, The Netherlands; 2 Department of Cardiology, Erasmus MC – University Medical Center Rotterdam, Rotterdam, The Netherlands; 3 Department of Epidemiology, Harvard T.H. Chan School of Public Health, Boston, MA USA; 4 Thorax Center, University Medical Center Groningen, Groningen, The Netherlands; 5 Department of Cardiology, University Medical Center Utrecht, Utrecht, The Netherlands

**Keywords:** Pacemaker longevity, Pacemaker follow-up, Generator replacement, Pacemaker infection, Pacemaker recall

## Abstract

**Aims:**

After decades of experience and strongly improved technology, service time of pacemaker generators is expected to increase. To test this hypothesis, we conducted a retrospective review of a large cohort of patients with a pacemaker.

**Methods:**

We reviewed data collected between 1984 and 2006 in the first national Dutch pacemaker registry. This registry covered 96% of all generators implanted. We analysed the time of and reason for explantation of pacemaker generators. A 7-year follow-up interval after first implantation and following replacements was used to analyse changes over time.

**Results:**

During 22 years of data collection, nearly 97,000 first pacemaker generators were implanted. A total of 27,937 (22.4%) generators were explanted within a mean of 6.3 (standard deviation 3.3) years. Reasons for approximately 60% of these explantations were ‘end of life’ of the pacemaker generator or elective system change. Complications or failures such as infections and recalls accounted for approximately 20% of the explantations. For the remaining 20%, the reasons for explantation had not been registered.

**Conclusion:**

Despite progress in technology, a substantial proportion of pacemaker generators is explanted before its expected service time, with one in five generators being replaced due to technical failures, infections or other complications. Furthermore, the time interval between pacemaker implantation and explantation due to normal ‘end of life’ (battery EOL) decreased. Infections continue to rank highly as a cause for pacing system replacement, despite all current preventive measures.

## Introduction

Chronic stimulation of the heart with pacemakers for bradycardia and other indications has been applied worldwide in increasing numbers [1, 2]. In the Netherlands, 3236 first pacemakers were implanted in 1984 (225 implants per million inhabitants) [3], while 10,389 pacemakers were implanted in 2011 (468/million inhabitants) [4].

Pacing devices have become technically more sophisticated to enable further options for sensing, pacing and monitoring, as well as sustainability for simultaneous use of other devices and techniques such as magnetic resonance imaging. At the same time, the devices got substantially smaller [5–7]. These developments required a more robust design of the device, IT facilities, and increased lifespan of the battery. The incremental need of remote monitoring to support an intense technical follow-up to optimise the care of the individual pacemaker recipient also required technical innovations.

Because previous surveys showed a substantial complication rate [5, 6, 8, 9], we aimed to investigate trends in the duration of service time or longevity of pacemaker generators after first implantation and re-implantation. For this purpose, we studied the reasons for replacement of pacemakers. We anticipated that growing experience, guidelines and clustering of treatment facilities would increase service time of the devices and complication rates would gradually diminish over several decades.

## Patients and methods

### Setting

Data were retrieved from the Central Pacemaker Patients Registration (CPPR) from the Netherlands Pacemaker Registry Foundation (CPPR-SPRN). The registry has been described in detail elsewhere [3, 10]. In brief: in 1982, CPPR-SPRN was established and the computerised registration began. Cardiologists and allied professionals were invited to voluntarily register data of each patient, pacemaker generator and leads on the former European pacemaker card. Data on symptoms, indication and diagnosis, brand of pacemaker and leads, type, follow-up visits, explantation, hospital transfer and death were registered according to European Registry Guidelines established in 1982 and later.

### Monitoring and validation of data

Until 1989, data were registered centrally; a carbon copy of the European pacemaker card had to be sent to the registry. From 1989 onwards, a digitalised registration was used with automatic communication between the central registration computer and the local computer of the implanting centre. During daily conversions into the database, multiple checks were performed on missing data, conformation with already stored information and plausibility [11]. Additionally, the data were periodically returned to the clinics for correction purposes. In 1997, a validation process was performed to obtain a better insight into the quality of the database. When the central registry was compared with patient files of participating hospitals and sales data from manufacturers, 95.7% of pacemaker generators could be retrieved [12].

### Cohort and outcome definition

Patients admitted for implantation of the first pacemaker between 01.01.1984 and 01.01.2006 were included in this nationwide cohort study. A total of 452 implantations were excluded because of inconsistencies in the registered data, e. g. a new implantation was registered after the supposed date of death of a patient, or the same pacemaker was registered more than once with different explantation dates. This resulted in a cohort with 96,900 patients having a first pacemaker implanted between January 1984 and January 2006, followed by 27,937 explantations (of which 27,659 replacement procedures) until January 2008. The years 2006 and 2007 were used for follow-up only. Additionally, for part of the analyses, the cohort was subdivided into three strata with 7 years of follow-up after each implantation, leaving 66,223 patients who received a first pacemaker between January 1984 and January 2001.

### Exposure

The primary interest was the number of pacemaker replacements or explantations and the reasons for these interventions. In this study ‘service time’ is defined as the time between pacemaker implantation and replacement or removal of the generator.

### Analysis

Analyses of explantations and replacements were performed on:

1. the entire cohort of patients (*n* = 96,900) having first implantations and re-implantations during the study period irrespective of available duration of follow-up, and
2. three strata (*n* = 66,223 patients) to identify changes over time.

Each implantation in these strata was followed for a maximum of 7 years or until explantation, whichever came first. For this purpose, first implantations and re-implantations during the years 1984–2000 could be used, while data from 2001–2007 were used for follow-up only. We chose a 7-year follow-up period because the mean duration of follow-up for explanted pacemaker generators falls within this time interval, as also observed by Hauser et al. [13]. Furthermore, with 66,223 first implantations during 1984–2000, two thirds of the cohort would remain available for analysis.* P*-values were calculated with chi-square analysis and independent samples* t* test.

### Sensitivity analysis

To estimate the proportion of deaths that was (voluntarily) registered in SPRN, we performed a sensitivity analysis by looking up patients from the Rotterdam Study, a large prospective cohort study on inhabitants of the Ommoord area in Rotterdam [14], in the SPRN database. First, we investigated on basis of gender and date of birth whether a participant from the Rotterdam Study was registered in SPRN. Subsequently, we validated each retrieval by using the pacemaker implant date. The date of death is registered for each participant of the Rotterdam Study.

## Results

Between 01.01.1984 and 01.01.2006, 96,900 patients received a first pacemaker. Approximately 53% of the patients were men and the mean age at time of first implantation was 72.9 years (standard deviation [SD] 13.0). Baseline characteristics are provided in Tab. 1.

**Table 1:** Baseline characteristics of patients in the nationwide CPPR-SPRN database having a first pacemaker implanted (*n* = 96,900), the Netherlands 1984–2007

	1984–1990	1991–1995	1996–2000	2001–2005	2006–2007	Total study period
**Patients** ^**a**^						
Mean age at first implantation, years (SD)	72.7 (13.0)	73.0 (13.0)	72.9 (13.2)	73.1 (12.9)	NA	72.9 (13.0)
Female gender, *n* (%)	11,628 (48.7)	9419 (47.9)	10,569 (46.6)	14,045 (45.8)	NA	45,661 (47.1)
Deaths, *n* (% of patients that received first pacemaker in this period)^b^	7245 (30.4)	6119 (31.1)	5849 (25.8)^c^	3721 (12.1)^c^	NA	22,934 (23.7)
Follow-up duration (range), years	17–23	12–16	7–11	2–6	NA	2–23
**Pacemaker generators**						
*Implantations, n (% of total* ^*b*^ * implanted pacemakers during study period), of which: *	24,952 (20.0)	23,451 (18.8)	29,789 (23.9)	41,569 (33.4)	4798 (3.9)	124,559 (100.0)
– First pacemaker	23,870 (95.7)	19,659 (83.8)^c^	22,694 (76.2)^c^	30,677 (73.8)^c^	NA	96,900 (77.8)
– First replacement	1004 (4.0)	3352 (14.3)^c^	5821 (19.5)^c^	8386 (20.2)	3571 (74.4)	22,134 (17.8)
– Second replacement	73 (0.3)	389 (1.7)^c^	1050 (3.5)^c^	1930 (4.6)^c^	908 (18.9)	4350 (3.5)
– Third or more replacement	5 (<0.1)	51 (0.2)^c^	224 (0.8)^c^	576 (1.4)^c^	319 (6.6)	1175 (0.9)
*Explantation of pacemakers implanted in this period, n (%), of which:*	7238 (29.0)	8402 (35.8)^c^	9250 (31.1)^c^	3000 (7.2)^c^	47 (1.0)	27,937 (22.4)
– Explantation <7 years, *n* (%)	3233 (44.7)	4489 (53.4)^c^	5745 (62.1)^c^	NA	NA	16,514 (59.1)
– Explantation <5 years, *n* (%)	1692 (23.4)	1972 (23.5)	2455 (26.5)^c^	NA	NA	8586 (30.6)
– Explantation <3 years, *n* (%)	950 (13.1)	943 (11.2)^c^	1196 (12.9)^c^	NA	NA	4683 (16.8)
Without replacement/without immediate replacement, *n* (%)	41 (0.6)	33 (0.4)	66 (0.7)	181 (6.0)^c^	11 (23.4)	332 (1.2)
*Mean duration of service time for explanted pacemakers, years (SD)*	7.7 (4.0)	6.8 (3.0)^c^	6.0 (2.4)^c^	2.9 (2.0)^c^	0.5 (0.5)	6.3 (3.3)
*Median duration service time for explanted pacemakers, years*	7.5	6.8	6.4	2.8	0.3	6.4

For 2006–2007 we used data on replacements only

*SD* standard deviation, *NA* not available

^a^For 75 patients, data on gender was missing. For 38 males and 22 females, data on age was missing

^b^Data on number of deaths is incomplete

^c^Significantly different compared to previous time interval (*p* ≤ 0.001), 2006–2007 not tested

### Pacemaker generator replacements and removals

During the study period, 22,134 patients (22.8%) had at least one pacemaker generator replacement or removal and 4350 patients (4.5%) had more than one. In total, 27,937 pacemaker generators were replaced or removed (22.4% of total number of implants), including 332 pacemaker generators that were coded as a removal without replacement, although it appeared that some of these patients did receive a new pacemaker after several weeks to months (Tab. 1). The mean duration of follow-up to pacemaker generator replacement or removal (service time) during the whole study period was 6.3 (SD 3.3) years. Approximately 60% of the explantations occurred within 7 years after implantation, 30.6% within 5 years and 16.8% within 3 years (Tab. 1).

Approximately 19% of the pacemaker generators were replaced or removed following device failure or complications and in 20% the reason for explantation was not available (Tab. 2). Analysis of pacing systems stratified for the period in which a pacemaker was implanted and followed for a maximum of 7 years shows that the percentage of explantations within 7 years due to infection lies between 4.0–4.5% for pacemaker generators and did not significantly change over time. The percentage of recalled pacemaker generators within 7 years of implantation increased during the study period, whereas the percentage of device failures significantly decreased towards the end of the study period from 3.9 to 1.8% of the pacemaker generator replacements (*p* ≤ 0.001; Tab. 2).

**Table 2:** Reasons for pacemaker generator replacements within 7 years of follow-up after implantation, stratified for implantation period, and for the entire cohort irrespective of duration of follow-up in the nationwide CPPR-SPRN database, the Netherlands 1984–2007

Reason for explantation of pacemaker generators within 7 years of follow-up, stratified for implantation period^a^ *n* (% of explanted pacemakers)				All pacemakers explanted during study period^b^
	1984–1990	1991–1995	1996–2000	1984–2007
Device failure (sensing, programming, output, rate, connector)	126 (3.9)	151 (3.4)	105 (1.8)^c^	509 (1.8)
Recall	32 (1.0)	174 (3.9)^c^	270 (4.7)	583 (2.1)
Infection	149 (4.6)	176 (3.9)	248 (4.3)	884 (3.2)
Other complication (mechanical protrusion, erosion, wound pain)	31 (1.0)	31 (0.7)	39 (0.7)	153 (0.5)
Elective for system change	319 (9.9)	450 (10.0)	554 (9.6)	2775 (9.9)
System change – Haemodynamic reasons	90 (2.8)	261 (5.8)^c^	398 (6.9)	1513 (5.4)
System change – Electrode problem	182 (5.6)	325 (7.2)	178 (3.1)^c^	1003 (3.6)
System change – other reasons	37 (1.1)	53 (1.2)	61 (1.1)	214 (0.8)
Normal ‘end of life’, of which:	908 (28.1)	1800 (40.1)^c^	2678 (46.6)^c^	14,077 (50.4)
– <7 years, *n* (% of all explantations <7 years^d^)	908 (28.1)	1800 (40.1)^c^	2678 (46.6)^c^	6349 (38.4)
– <5 years, *n* (% of all explantations <5 years^d^)	202 (11.9)	393 (19.9)^c^	587 (23.9)	1759 (20.5)
– <3 years, *n* (% of all explantations <3 years^d^)	29 (3.1)	48 (5.1)	87 (7.3)	307 (6.6)
Premature ‘end of life’	77 (2.4)	140 (3.1)	126 (2.2)	520 (1.9)
Reason uncoded, unknown or unspecified	1282 (39.6)	928 (20.7)^c^	1088 (18.9)	5706 (20.4)
Total	3233 (100.0)	4489 (100.0)	5745 (100.0)	27,937 (100.0)

^a^For each stratum, the duration of follow-up was maximised at 7 years. Hence, the years 2001–2007 were used for follow-up only. Consequently, numbers of the first three columns do not add up to the numbers in the fourth column

^b^Regardless duration of follow-up

^c^Significantly different compared to previous time interval (*p* ≤ 0.001)

^d^See Tab. 1

A total of 50.4% of pacemaker generators were explanted because of normal ‘end of life’ of the generator (Tab. 2). For these generators, the service time varied widely. When compared to the number of explantations within different periods of follow-up, 38.4% of the generators was explanted for normal ‘end of life’ within <7 years, 20.5% <5 years, and 6.6% <3 years. These percentages increased over time (Tab. 2).

Dual chamber systems were significantly more often explanted for normal ‘end of life’ <5 years and <7 years and technical reasons than single chamber systems (Tab. 3). Overall, dual chamber systems were significantly more often explanted than single chamber systems (*p* ≤ 0.001; Tab. 3).

**Table 3:** Comparison of specific replacement or explantation reasons between first pacemakers with NASPE codes VVI/VVIR and DDD/DDDR in the nationwide CPPR-SPRN database, the Netherlands 1984–2007

	VVI/VVIR		DDD/DDDR		*p*
	*n*	%	*n*	%	
Number of first implantations of this pacemaker type^a^	47,945	49.5	38,060	39.3	–
Number of explantations of first pacemaker of this type	4391	9.2	5861	15.4	<0.001
**Reason for explantation**					
Recall	141	3.2	209	3.6	0.328
Complication	226	5.1	350	6.0	0.073
Failure	161	3.7	154	2.6	0.003
Premature ‘end of life’	123	2.8	164	2.8	0.993
*Normal ‘end of life’*					
<7 years	1302	29.7	2716	46.3	<0.001
<5 years	273	6.2	690	11.8	<0.001
<3 years	53	1.2	46	0.8	0.408

*NASPE* North American Society of Pacing and Electrophysiology

^a^% compared to all first pacemaker implantations between 01.01.1984 and 01.01.2006, *n* = 96,900

### Sensitivity analysis

A total of 258 participants of the Rotterdam Study were found in the SPRN database. During the study period, 148 (57.4%) died. Of these deaths, 60 (40.5%) were also registered in SPRN. We consider 92% of these registrations to be accurate (within 3 months from the registered date of death in the Rotterdam Study). Age at death and implanted pacemaker type did not statistically significantly differ between the group of patients registered as deceased and the group of patients not registered as deceased in SPRN (*p* = 0.56 and *p* = 0.90, respectively).

## Discussion

Our results show that 22% of pacemaker generators were replaced or removed at least once between 1984 and 2008. Approximately one in five pacemaker generators were explanted due to technical failures or complications during 20 years of follow-up. Complication and failure rates for pacemaker generators did not improve during at least the first 15 years of the registry. Furthermore, we found that explantation of pacemaker generators for normal ‘end of life’ occurred at a decreasing follow-up time. The explantation rate found in the Danish Pacemaker Registry, which covers the same period, compares to ours [15].

‘Normal service time’ of pacemaker generators includes the lifespan of the pacemaker generator in terms of longevity of the battery and of the electronic components. Time intervals between pacemaker implantation and removal vary from a mean of 6.8 years for dual chamber devices and 9.7 years for single chamber devices in one study [16] and 7.3 years found in studies on several types and brands of devices [13, 17]. Kindermann et al. found a median time interval to battery depletion of 8.2 years [18]. However, cohort size, study duration, number of participating hospitals and number and type of different pacemakers differed between these studies and differed compared with ours.

More ancillary functions and operational algorithms than standard pacing, sensing and communicating with the programmer, require more battery capacity and may thus affect service time. This may cause newer models to offer shorter service time than expected [17, 19]. Hauser et al. restricted service time to the battery life time ending with the appearance of the elective replacement indicator, considering a longevity of >3 years after implant as a minimal requirement [13]. They found that the average pulse generator was implanted for 7.3 years (SD 3.1). The almost twofold increase of the most frequently registered reason for pacemaker generator replacement in our study – normal ‘end of life’ – suggests that battery longevity did not improve during at least the first 15 years of the study. This underscores the need for longer service time of pacemaker generators by new battery technology that permits pacing for at least 10 years. A longer ‘normal service time’ of pacemaker generators would be more than welcome because replacement of pacemakers exposes patients to the risk of device infection.

Nearly one in five pacemaker generators was explanted following a complication or failure in our study. A review of reports submitted to the FDA and analysis of device registries published in literature show that pacemaker generator failures included acute or premature battery depletion, connector malfunctions, electrical problems such as short circuit, inappropriate high-current drains, or hermetic seal abnormalities. Such complications sometimes cause major clinical events [5, 6, 13]. Similar technical failures also emerged in our registry: premature ‘end of life’, electrode problems, recalls and device failures accounted for more than 9% of the replacements of pacemaker generators in our study. Furthermore, the percentage of explantations following a recall increased over time in parallel with medical device regulation and post-marketing surveillance. Nevertheless, studies have shown that, despite increasing complexity of the components of pacemaker generators over the past decades, the overall replacement rate for technical failures dropped [5, 6]. However, technical defects may remain unnoticed despite regular follow-up, as a previous study implies [20]. This post-mortem study of pacemaker generators demonstrated that in 3.8% of patients, deceased after an average of 4 years of pacing, a life-threatening technical failure was present and in 3.0% a potentially life-threatening technical failure that may have caused their death [20]. The sensitivity analysis on the Rotterdam Study sub cohort showed that 50% of the patients died within 3 years after implantation of the last registered pacemaker. We cannot rule out that a proportion of these patients died following pacemaker malfunction.

### Limitations

In nearly 20% of the pacemaker generator replacements, the reason and time for replacement or removal remained unknown. Missing data could be ascribed to the voluntary participation in the registration; some hospitals (<5%) did not register data or did not register during the entire study period. Data were provided by each participating hospital individually. This may have led to differences in interpretation of the requested information. Relevant variables such as information on comorbidity, medication use, and cardiac function were not recorded at all. This precluded us from adjusting for potential clinical confounders. Furthermore, the registry did not include information on pacemaker setting, pacing threshold and lead impedance. These factors are known to influence battery longevity [17, 18].

Finally, detailed information on the proportion of patients that may have been lost to follow-up is unknown. Information on date of death was voluntarily registered and in 10% of the reported deaths no date of death was provided. Sensitivity analysis showed that approximately 60% of the deaths could be missing.

### Conclusions

A substantial proportion of pacemaker generators is explanted before its expected service time, with one in five generators being replaced due to technical failures, infections or complications. Furthermore, the time interval between pacemaker implantation and explantation due to normal ‘end of life’ decreased. Our observations underscore the need to program only the pacemaker generator features that have proven clinical benefit to avoid reductions of the service time of the device. The results of our study indicate that a continued highly detailed registry to identify risk factors for premature replacement is needed to maximise the service time of pacemakers.
